# Cancer risk in HIV patients with incomplete viral suppression after initiation of antiretroviral therapy

**DOI:** 10.1371/journal.pone.0197665

**Published:** 2018-06-05

**Authors:** Jennifer S. Lee, Stephen R. Cole, Chad J. Achenbach, Dirk P. Dittmer, David B. Richardson, William C. Miller, Christopher Mathews, Keri N. Althoff, Richard D. Moore, Joseph J. Eron, for the Center for AIDS Research (CFAR) Network of Integrated Clinical Systems (CNICS)

**Affiliations:** 1 Department of Epidemiology, University of North Carolina at Chapel Hill, Chapel Hill, North Carolina, United States of America; 2 Department of Epidemiology, Johns Hopkins University, Baltimore, Maryland, United States of America; 3 Center for AIDS Research, University of North Carolina at Chapel Hill, Chapel Hill, North Carolina, United States of America; 4 Department of Medicine, Division of Infectious Diseases, Center for Global Health, Feinberg School of Medicine, Northwestern University, Chicago, Illinois, United States of America; 5 Department of Microbiology and Immunology, Lineberger Comprehensive Cancer Center, University of North Carolina at Chapel Hill, Chapel Hill, North Carolina, United States of America; 6 Division of Epidemiology, The Ohio State University, Columbus, Ohio, United States of America; 7 University of California, San Diego, California, United States of America; 8 Department of Medicine, Johns Hopkins University, Baltimore, Maryland, United States of America; 9 Department of Medicine, University of North Carolina at Chapel Hill, Chapel Hill, North Carolina, United States of America; University of Pittsburgh Centre for Vaccine Research, UNITED STATES

## Abstract

**Background:**

Cancer causes significant morbidity and mortality among HIV patients in the US due to extended life expectancy with access to effective antiretroviral therapy. Low, detectable HIV RNA has been studied as a risk factor for adverse health outcomes, but its clinical impact on cancer risk remains unclear. The objective of this study was to determine whether HIV RNA <1,000 copies/mL six months after starting therapy was associated with 10-year first cancer risk.

**Methods:**

We followed 7,515 HIV therapy initiators from a US-based multicenter clinical cohort from 1998 to 2014. We used nonparametric multiple imputation to account for viral loads that fell below assay detection limits, and categorized viral loads six months after therapy initiation into four groups: <20, 20–199, 200–999, and >999 copies/mL. We calculated estimates of the cumulative incidence of cancer diagnosis, accounting for death as a competing event. Inverse probability of exposure and censoring weights were used to control for confounding and differential loss to follow up, respectively.

**Results:**

Crude 10-year first cancer risk in the study sample was 7.03% (95% CI: 6.08%, 7.98%), with the highest risk observed among patients with viral loads between 200 and 999 copies/mL six months after ART initiation (10.7%). After controlling for baseline confounders, 10-year first cancer risk was 6.90% (95% CI: 5.69%, 8.12%), and was similar across viral load categories.

**Conclusion:**

Overall risk of first cancer was not associated with incomplete viral suppression; however, cancer remains a significant threat to HIV patients after treatment initiation. As more HIV patients gain access to treatment in the current “treat all” era, occurrences of incomplete viral suppression will be observed more frequently in clinical practice, which supports continued study of the role of low-level HIV RNA on cancer development.

## Introduction

Effective antiretroviral therapy (ART) suppresses human immunodeficiency virus (HIV) levels to below the detection limits of assays used in clinical practice in the United States. Uptake of ART has resulted in lower incidence of acquired immune deficiency syndrome (AIDS)-defining illnesses, prolonged survival, and rising incidence of non-AIDS-defining cancers and chronic diseases among people living and aging with HIV [1]. Cancer is the second-leading cause of death among both men and women in the US [2], and is also a significant cause of morbidity and mortality among HIV patients [1, 3]. The risk of developing specific cancers may be higher among people infected with HIV compared to the general population due to immunosuppression, oncogenic viral coinfections, and elevated prevalence of certain risk behaviors, such as smoking and alcohol abuse [4–6].

Not all HIV patients on treatment achieve and maintain undetectable viral loads, and the impact of low levels of detectable HIV ribonucleic acid (RNA) under 1,000 copies/mL on the risk of comorbid disease, such as cancer, remains unclear. Incomplete viral suppression appears to increase the risk of certain cancers [7–9], but the association between early virologic control and cancer risk has not been rigorously evaluated. Optimally, HIV patients initiating ART would achieve undetectable HIV RNA within six months [10], and here we explore whether failure to achieve this milestone is associated with cancer risk. Because low, detectable HIV RNA may be associated with ongoing inflammation [11, 12], it is biologically plausible that incomplete viral suppression may have predictive value in assessing the long-term risk of developing various cancers, particularly those associated with chronic inflammation and viral coinfection [13, 14]. Failure to suppress HIV RNA after initiation of ART may also be a marker of suboptimal adherence that could influence long-term clinical outcomes. The objective of this study is to examine 10-year first cancer risk among HIV patients with a single low-level viral load measurement under 1,000 copies/mL collected six months after ART initiation, while accounting for death as a competing risk.

## Methods

### Study population

We used data from the Center for AIDS Research (CFAR) Network of Integrated Clinical Systems (CNICS), a multicenter clinical cohort of over 30,000 HIV patients in the United States. CNICS maintains a clinical data repository from electronic medical record systems to support HIV research [15]. The CNICS cohort includes patients aged 18 years and older who initiated primary care in or after January 1995 at one of eight CFAR sites: Case Western Reserve University; Fenway Community Health Center of Harvard University; Johns Hopkins University; University of Alabama at Birmingham; University of California, San Diego; University of California, San Francisco; University of North Carolina at Chapel Hill; and University of Washington. CNICS is a dynamic cohort, with approximately 1,400 new patients enrolling and 10% of patients leaving care annually [15].

Upon entry into CNICS, demographic and historical information, including prior diagnoses and antiretroviral treatment, was collected. After enrollment, patient data were prospectively captured at clinic visits and include prescribed medications, laboratory test results, and conditions diagnosed by providers. CNICS participants were typically seen in clinical care every three to four months, though frequency of follow-up was patient specific. The research of CNICS was approved by the Office of the Institutional Review Board for Human Use at the University of Alabama at Birmingham. All participating study sites received consent from their local institutional review boards to conduct the research activities of CNICS. The study conducted here, a secondary analysis of extant, de-identified CNICS data, was reviewed by the Office of Human Research Ethics at the University of North Carolina at Chapel Hill, which determined that it did not constitute human subjects research.

A total of 27,865 patients entered the CNICS cohort between 1 January 1998 and 31 December 2013. We excluded patients who initiated monotherapy or dual therapy prior to or with no history of starting combination ART (defined as three or more ART drugs prescribed concurrently) (n = 3,499), initiated ART prior to entering CNICS (n = 6,405), initiated ART after 31 December 2013 (n = 282), or had no history of initiating ART (n = 4,067). We excluded patients who were diagnosed with cancer prior to ART initiation (n = 570), were diagnosed with cancer within six months of starting ART (n = 151), died within six months of starting ART (n = 99), or did not have at least one viral load measurement six months (-30/+90 days) after ART initiation (n = 3,285). Patients with missing covariate data (missing race/ethnicity information [n = 88], no recorded CD4 count six months [-30/+90 days] after ART initiation [n = 308], no pre-ART viral load measurement collected between 60 days prior to CNICS entry and ART initiation [n = 261]) or pre-ART viral load measurements that suggested unrecorded prior exposure to treatment (<1,000 copies/mL) (n = 1,335) were also excluded. The final study sample comprised 7,515 patients.

### Viral load assessment

The exposure of interest was HIV RNA six months (-30/+90 days) after ART initiation. For patients who had more than one eligible viral load measurement during the specified 120-day window, we used the measurement that was closest to six months after date of ART initiation. Viral load measurements were determined by quantitative amplification assays and expressed as the number of HIV copies per milliliter of blood plasma (copies/mL). Viral load assay sensitivity varied over time and by CNICS site; the lower limits of detection for the most commonly used assays were 20, 30, 40, 48, 50, 75, and 400 copies/mL. Observed viral load measurements six months after ART initiation ranged from 6 to over 4 million copies/mL.

### Endpoint ascertainment

The outcome of interest was time from six months after ART initiation to diagnosis of first invasive cancer, excluding nonmelanoma skin cancer. All cancer cases diagnosed through 31 December 2014 and recorded at CNICS sites were verified by medical record review [16]. Cancer data collected by CNICS included date of diagnosis, tumor site, diagnosis method (histopathology, clinical exam, radiography, or historical information), histology, stage, and grade.

Death from any cause was considered a competing risk in the analysis. National Death Index and state death certificate records were queried regularly by all CNICS sites to confirm recorded dates of deaths.

### Statistical analysis

The start of follow-up for each patient was six months after date of ART initiation. Patients were followed until the earliest of the following: first cancer diagnosis, death, or loss to follow-up (defined as no recorded clinic visit, laboratory result, or hospitalization for 18 months). Death from any cause without a cancer diagnosis was considered a competing event. Data were administratively censored after 10 years or on 31 December 2014.

We used a proportional subdistribution hazards model [17] to compute nonparametric estimates of the cumulative incidence function of being diagnosed with incident cancer in the presence of the competing risk of death. We calculated 10-year risk differences and risk ratios and constructed risk curves stratified by viral load category [18]. We drew 200 nonparametric bootstrap samples with replacement from the original study population to estimate standard errors.

The majority of viral load observations included in our analyses were reported to be below specified lower limits of detection (i.e., 20, 30, 40, 48, 50, 75, and 400 copies/mL). For the original study population and each bootstrap sample, we used a nonparametric imputation approach with a censoring score model to account for left-censored viral load data [19]. For each viral load observation, we used logistic regression to estimate the conditional probability of left censoring given age, sex, race/ethnicity, sexual identity, injection drug use, CD4 count, clinical AIDS status, year of ART initiation, ART regimen, pre-ART viral load, chronic hepatitis status, statin use, smoking status, at-risk alcohol use, CNICS site, death, and incident cancer [20]. Restricted quadratic splines were used to model age and CD4 count, with knots at the 5^th^, 35^th^, 65^th^, and 95^th^ percentiles [21]. We computed nonparametric maximum likelihood estimates [22, 23] of the distribution function of viral load, stratified by quintiles of the predicted probability of being left censored. These estimates were used to impute left-censored viral load observations, and imputed viral load values were bounded between zero and the detection limit of the viral load assay used. Thirty imputed datasets were generated for the original study population and each bootstrap sample.

Patients were assigned to the following exposure categories based on their observed or imputed viral load at baseline (six months after ART initiation): <20, 20 to 199, 200 to 999, and >999 copies/mL. Because the US Department of Health and Human Services and the AIDS Clinical Trials Group currently define virologic failure as one confirmed viral load measurement at or above 200 copies/mL [10], we divided low-level viral loads under 1,000 copies/mL into two categories, 20 to 199 copies/mL, and 200 to 999 copies/mL. The reference category was viral load <20 copies/mL.

We used inverse probability of exposure weights [24, 25] to control for differences at baseline among patients across the four viral load categories and calculate cumulative incidence estimates standardized to the total study population. Sex, race/ethnicity, sexual identity, and injection drug use were assessed at entry into the CNICS cohort. Pre-ART viral load, ART regimen, and year of ART initiation were assessed at ART initiation. Age, CD4 count, clinical AIDS status, chronic hepatitis status, statin use, smoking status, at-risk alcohol use, and CNICS site were assessed at study baseline. Restricted quadratic splines were used to model age and CD4 count. Using a multinomial logistic regression model, we estimated the conditional probability of having a viral load in each viral load category.

Additionally, we estimated inverse probability of censoring weights to account for potentially informative loss to follow-up by viral load category. The inverse probability of exposure and inverse probability of censoring weights were stabilized, and the product of the stabilized weights had a mean of 1.0 in the imputed datasets of the original study sample, with a minimum of 0.12 and maximum of 13. We used SAS version 9.4 (SAS Institute Inc., Cary, NC) for analyses, and R version 3.3.1 (R Foundation for Statistical Computing, Vienna, Austria) for figures.

## Results

We identified 7,515 CNICS patients who met our study inclusion criteria (Table 1). Median age at baseline (six months after ART initiation) was 39 (interquartile range [IQR]: 32, 46) years, 82% were male, 44% were white, 37% were black/African American, 61% identified as men who have sex with men, and 12% reported having ever injected drugs. Median pre-ART viral load was 73,320 (IQR: 22,000, 234,048) copies/mL, median year of ART initiation was 2007 (IQR: 2003, 2010), and median CD4 count was 356 (IQR: 201, 537) cells/mm^3^. At baseline, 26% of study patients had been diagnosed with AIDS, 40% had been prescribed a protease inhibitor (PI)-based regimen, and 48% were prescribed a non-nucleoside reverse transcriptase inhibitor (NNRTI)-based regimen. Patients were followed for a median of 4.9 (IQR: 2.7, 8.1) years, for a total of 40,110 person-years. We recorded 290 cancer diagnoses during the study period, and 1,731 (23%) patients were lost to follow up.

**Table 1:** Demographic, clinical, and behavioral characteristics of 7,515 CNICS patients six months after ART initiation, averaged over 30 imputations, between 1 July 1998 and 30 June 2014.

Characteristic	Total n = 7,515	<20 cpm n = 4,281	20–199 cpm n = 1,694	200–999 cpm n = 393	>999 cpm n = 1,147
	No. (%)	No. (%)	No. (%)	No. (%)	No. (%)
Age, years ^a^	39 (32, 46)	39 (32, 47)	40 (33, 46)	40 (34, 47)	39 (32, 45)
Male ^b^	6,180 (82.2)	3,553 (83.0)	1,421 (82.5)	324 (80.7)	882 (76.9)
Race/ethnicity ^b^					
White, non-Hispanic	3,343 (44.5)	2,008 (46.9)	759 (44.8)	160 (40.7)	416 (36.3)
Black, non-Hispanic	2,800 (37.3)	1,426 (33.3)	627 (37.0)	181 (46.0)	566 (49.3)
Other, non-Hispanic	375 (5.0)	238 (5.6)	78 (4.6)	16 (4.2)	43 (3.7)
Hispanic	997 (13.3)	609 (14.2)	230 (13.6)	36 (9.1)	122 (10.6)
MSM, ever ^b^	4,611 (61.4)	2,753 (64.3)	1,037 (61.2)	224 (56.9)	597 (52.0)
IDU, ever ^b^	938 (12.5)	451 (10.5)	205 (12.1)	67 (17.1)	215 (18.7)
Smoking, ever	2,474 (32.9)	1,366 (31.9)	555 (32.8)	129 (32.8)	424 (37.0)
At-risk alcohol use, ever	1,125 (15.0)	609 (14.2)	244 (14.4)	58 (14.6)	214 (18.7)
Pre-ART viral load, cpm ^a^^,^^c^	73,320 (22,000; 234,048)	55,133 (17,296; 166,966)	109,225 (35,253; 349,189)	103,356 (35,573; 427,215)	93,071 (30,122; 299,230)
Year of ART initiation ^a^^,^^c^	2007 (2003, 2010)	2008 (2004, 2010)	2006 (2002, 2009)	2006 (2002, 2009)	2005 (2001, 2008)
ART regimen ^c^					
NNRTI-based	3,570 (47.5)	2,335 (54.5)	691 (40.8)	136 (34.6)	409 (35.7)
PI-based	2,997 (39.9)	1,420 (33.2)	782 (46.2)	209 (53.2)	586 (51.1)
INSTI-based	405 (5.4)	290 (6.8)	75 (4.4)	10 (2.5)	30 (2.6)
Other	543 (7.2)	236 (5.5)	147 (8.7)	38 (9.6)	122 (10.6)
CD4 count, cells/mm^3^ ^a^	356 (201, 537)	412 (254, 581)	338 (197, 511)	299 (177, 456)	204 (75, 362)
Clinical AIDS diagnosis	1,985 (26.4)	918 (21.4)	521 (30.7)	130 (33.1)	417 (36.4)
Chronic hepatitis B	193 (2.6)	82 (1.9)	50 (3.0)	11 (2.9)	49 (4.3)
Chronic hepatitis C	612 (8.1)	307 (7.2)	135 (8.0)	34 (8.8)	135 (11.8)
Statin use, ever	236 (3.1)	158 (3.7)	45 (2.6)	11 (2.8)	22 (1.9)

Abbreviations: AIDS, acquired immunodeficiency syndrome; ART, antiretroviral therapy; CNICS, Center for AIDS Research Network of Integrated Clinical Systems; cpm, copies per milliliter; INSTI, integrase strand transfer inhibitor; IDU, injection drug use; IQR, interquartile range; MSM, men who have sex with men; NNRTI, non-nucleoside reverse transcriptase inhibitor; PI, protease inhibitor.

^a^ Median (interquartile range).

^b^ Assessed at entry into CNICS cohort.

^c^ Assessed at ART initiation.

Of the 7,515 patients included in the study, 68% had viral loads six months after ART initiation that were below assay detection limits. After imputation, 56% of viral loads were under 20 copies/mL, 23% were between 20 and 199 copies/mL, 5% were between 200 and 999 copies/mL, and 15% were over 999 copies/mL. Plots of the distribution of viral load comparing our nonparametric multiple imputation approach to ad hoc substitution (replacing left-censored viral load observations with half of the detection limit of the viral load assay) are shown in S1 Fig.

Patients differed across viral load categories at baseline (Table 1). Patients with suppressed viral loads <20 copies/mL six months after ART initiation were more likely to be white, identify as men who have sex with men, have higher CD4 counts, have lower pre-ART viral loads, have started ART in the latter half of the study period, have been prescribed an NNRTI-based regimen, and have been prescribed statins. Patients with viral loads <20 copies/mL six months after starting ART were less likely to report injection drug use, have been prescribed a PI-based regimen, have chronic hepatitis, report having ever smoked, or report at-risk alcohol use.

The most common cancers observed in our study population were non-Hodgkin lymphoma (n = 39 cases, or 13.4% of all cancer cases), Kaposi sarcoma (n = 37; 12.8%), lung cancer (n = 30, 10.3%), Hodgkin lymphoma (n = 26; 9.0%), prostate cancer (n = 22; 7.6%), anal cancer (n = 18; 6.8%), breast cancer (n = 14; 4.8%), and liver cancer (n = 14; 4.8%) (Table 2). Crude cancer risk in the study sample was 7.03% (95% confidence interval [CI]: 6.08%, 7.98%) (Table 3). The highest crude cancer risk was observed among patients with viral loads between 200 and 999 copies/mL six months after ART initiation (10.7%), with risk of cancer diagnosis ranging from 6.60% to 7.67% in the other three viral load categories.

**Table 2:** Number (%) of cancers observed in 7,515 CNICS patients, averaged over 30 imputations (rounded to nearest integer).

	Total n = 7,515	<20 cpm n = 4,281	20–199 cpm n = 1,694	200–999 cpm n = 393	>999 cpm n = 1,147
All cancers	290 (100)	152 (100)	62 (100)	22 (100)	54 (100)
Non-Hodgkin lymphoma	39 (13.4)	17 (11.2)	11 (17.7)	2 (9.1)	9 (16.7)
Kaposi sarcoma	37 (12.8)	17 (11.2)	5 (8.1)	3 (13.6)	12 (22.2)
Lung cancer	30 (10.3)	18 (11.8)	6 (9.7)	1 (4.5)	5 (9.3)
Hodgkin lymphoma	26 (9.0)	18 (11.8)	5 (8.1)	1 (4.5)	2 (3.7)
Prostate cancer	22 (7.6)	12 (7.9)	8 (12.9)	1 (4.5)	1 (1.9)
Anal cancer	18 (6.8)	8 (5.3)	5 (8.1)	3 (13.6)	4 (7.4)
Breast cancer	14 (4.8)	10 (6.6)	3 (4.8)	0	1 (1.9)
Liver cancer	14 (4.8)	7 (4.6)	2 (3.2)	1 (4.5)	4 (7.4)
Skin cancer (melanoma)	11 (3.8)	4 (2.6)	3 (4.8)	1 (4.5)	3 (5.6)
Oral cavity and pharyngeal cancer	10 (3.4)	5 (3.3)	1 (1.6)	1 (4.5)	3 (5.6)
Kidney cancer	8 (2.8)	4 (2.6)	1 (1.6)	2 (9.1)	1 (1.9)
Colon cancer	6 (2.3)	4 (2.6)	2 (3.2)	0	0
Leukemia	6 (2.3)	3 (2.0)	1 (1.6)	0	2 (3.7)
Laryngeal cancer	5 (1.7)	3 (2.0)	2 (3.2)	0	0
Multiple myeloma	5 (1.7)	2 (1.3)	2 (3.2)	0	2 (3.7)
Cervical cancer	4 (1.4)	2 (1.3)	1 (1.6)	0	1 (1.9)
Esophageal cancer	3 (1.0)	2 (1.3)	1 (1.6)	0	0
Uterine cancer	3 (1.0)	2 (1.3)	1 (1.6)	0	0
Thyroid cancer	3 (1.0)	2 (1.3)	0	1 (4.5)	0
Brain and nervous system cancer	2 (0.7)	2 (1.3)	0	0	0
Testicular cancer	2 (0.7)	2 (1.3)	0	0	0
Rectal and rectosigmoid cancer	2 (0.7)	1 (0.7)	1 (1.6)	0	0
Peritoneal & retroperitoneal cancer	2 (0.7)	1 (0.7)	0	0	1 (1.9)
Bladder cancer	1 (0.3)	1 (0.7)	0	0	0
Ovarian cancer	1 (0.3)	1 (0.7)	0	0	0
Soft tissue cancer	1 (0.3)	1 (0.7)	0	0	0
Stomach cancer	1 (0.3)	1 (0.7)	0	0	0
Vaginal cancer	1 (0.3)	0	1 (1.6)	0	0
Vulvar cancer	1 (0.3)	0	0	1 (4.5)	0
Pancreatic cancer	1 (0.3)	0	0	0	1 (1.9)
Small intestine cancer	1 (0.3)	0	0	0	1 (1.9)
Other (unspecified site)	8 (2.8)	3 (2.0)	1 (1.6)	3 (13.6)	1 (1.9)

Abbreviation: CNICS, Center for AIDS Research Network of Integrated Clinical Systems; cpm, copies per milliliter.

**Table 3:** Crude and standardized 10-year cumulative incidence, risk difference, and risk ratio estimates for first cancer diagnosis in 7,515 CNICS patients, averaged over 30 imputations.

	No. of events	No. of patients	Person- years	Crude			Standardized ^a^		
				Risk, % (95% CI)	RD, % (95% CI)	RR (95% CI)	Risk, % (95% CI)	RD, % (95% CI)	RR (95% CI)
Total	290	7,515	40,110	7.03 (6.08, 7.98)			6.90 (5.69, 8.12)		
<20 cpm	152	4,281	22,392	6.60 (5.34, 7.86)	0	1	6.76 (5.12, 8.39)	0	1
20 to 199 cpm	62	1,694	9,625	6.71 (5.25, 8.17)	0.10 (-1.74, 1.94)	1.02 (0.73, 1.30)	6.88 (5.08, 8.68)	0.12 (-2.08, 2.33)	1.02 (0.68, 1.36)
200 to 999 cpm	22	393	2,124	10.7 (5.74, 15.6)	4.08 (-0.92, 9.08)	1.62 (0.83, 2.40)	6.82 (3.50, 10.1)	0.06 (-3.73, 3.86)	1.01 (0.43, 1.59)
>999 cpm	54	1,147	5,969	7.67 (5.31, 10.0)	1.07 (-1.69, 3.84)	1.16 (0.72, 1.61)	7.44 (4.10, 10.8)	0.68 (-3.05, 4.41)	1.10 (0.53, 1.67)

Abbreviations: CI, confidence interval; CNICS, Center for AIDS Research Network of Integrated Clinical Systems; cpm, copies per milliliter; RD, risk difference; RR, risk ratio.

^a^ Standardized estimates controlled for age, sex, race/ethnicity, male-to-male sexual contact, injection drug use, smoking, at-risk alcohol use, pre-ART viral load, year of ART initiation, ART regimen, CD4 count, clinical AIDS status, chronic hepatitis status), statin use, and study site.

**Fig 1:**
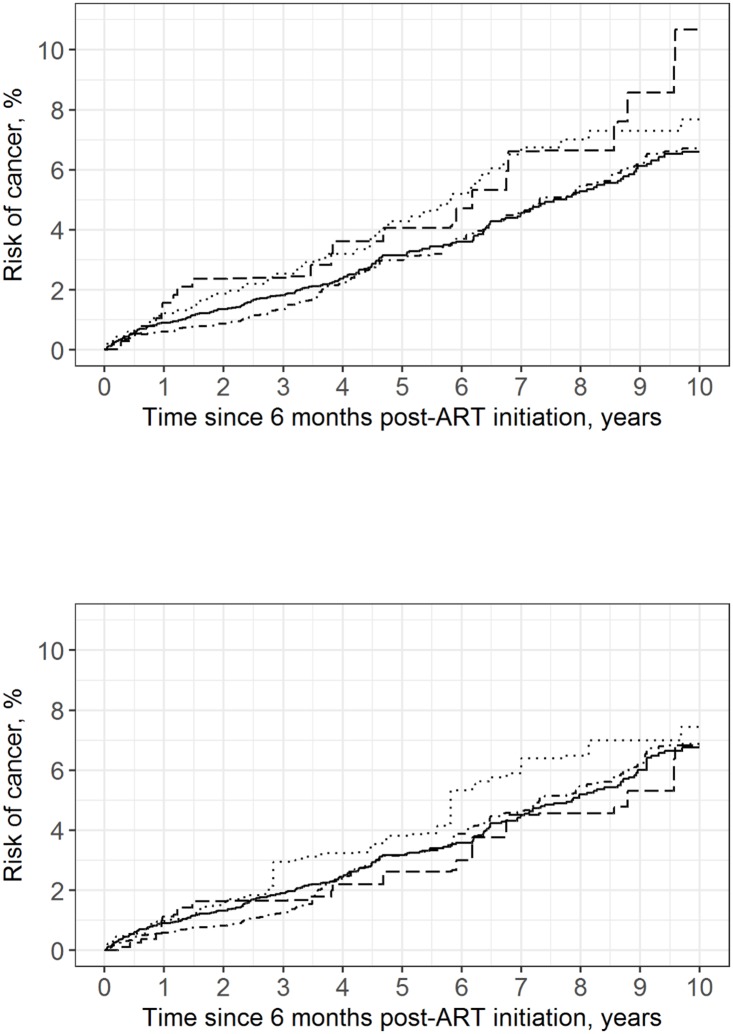
Crude and standardized risk curves for first cancer diagnosis in 7,515 CNICS patients, stratified by viral load category, averaged over 100 imputations. Standardized estimates controlled for age, sex, race/ethnicity, male-to-male sexual contact, injection drug use, smoking, at-risk alcohol use, pre-ART viral load, year of ART initiation, ART regimen, CD4 count, clinical AIDS status, chronic hepatitis status, statin use, and study site. Solid line represents viral loads <20 copies/mL, dot-dashed line represents viral loads between 20 and 199 copies/mL, dashed line represents viral loads between 200 and 999 copies/mL, dotted line represents viral loads >999 copies/mL. (a) Crude; (b) Standardized.

After controlling for baseline characteristics, overall 10-year cancer risk was 6.90% (95% CI: 5.69%, 8.12%), with little variation in cancer risk by viral load category (range: 6.76% to 7.44%). The cumulative cancer incidence estimate for patients with viral loads between 200 and 999 copies/mL was markedly reduced after adjustment (crude risk of 10.7% vs. standardized risk of 6.82%); race/ethnicity, year of ART initiation, ART regimen, baseline CD4 count, and study site accounted for 75% of the change in estimate in this viral load category. Among patients with viral loads between 200 and 999 copies/mL six months after ART initiation who were diagnosed with cancer, 62% were black (compared to 47% of all cases in the total study population), 53% started ART between 1998 and 2000 (vs. 21%), 63% had been prescribed a PI-based regimen (vs. 46%), and 48% had a CD4 count of less than 200 cells/mm^3^ six months after starting therapy (vs. 36%). Crude and standardized risk curves for 10-year cumulative cancer incidence are shown in Fig 1.

**Fig 2:**
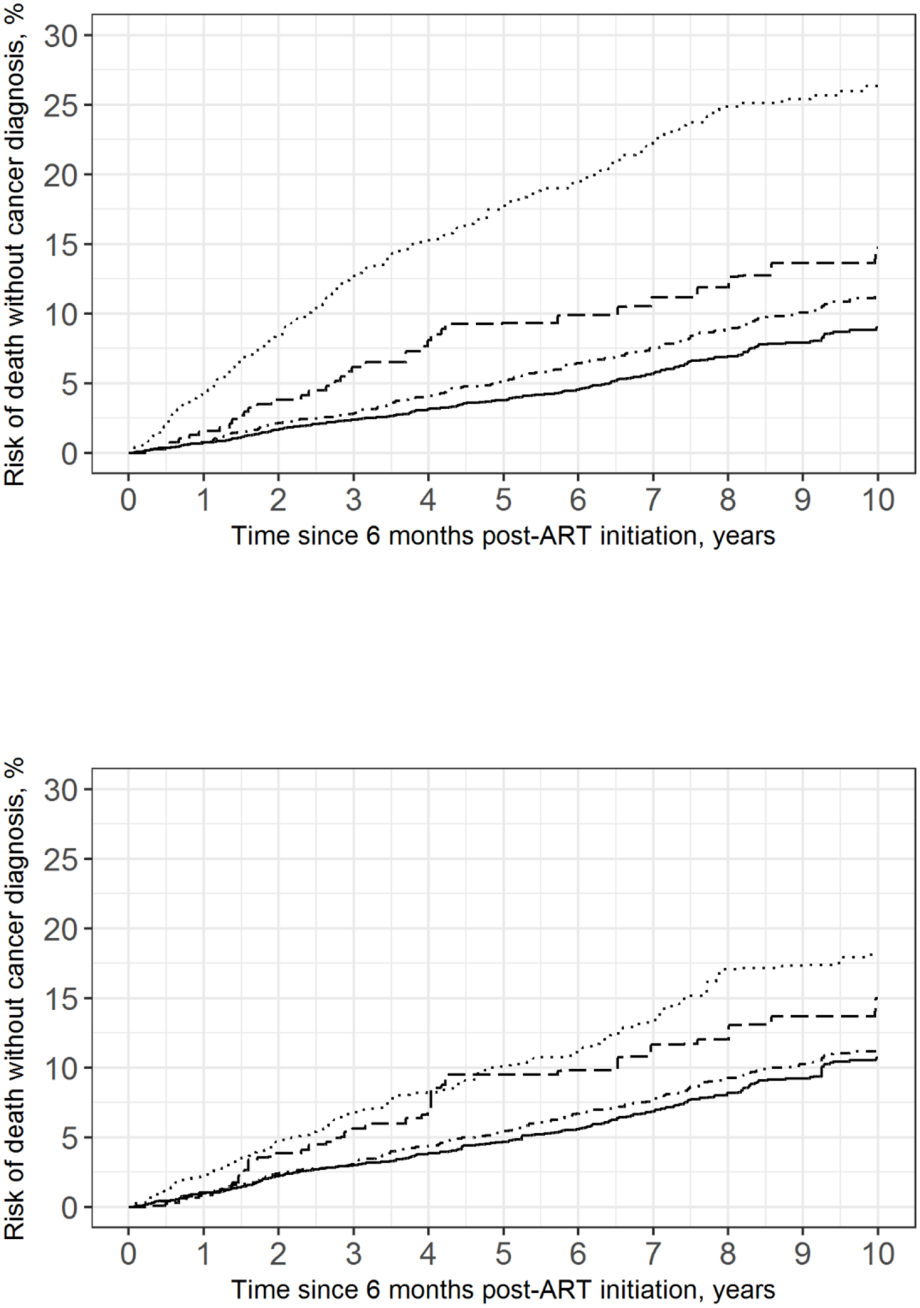
Crude and standardized risk curves for death without a cancer diagnosis in 7,515 CNICS patients, stratified by viral load category, averaged over 100 imputations. Standardized estimates controlled for age, sex, race/ethnicity, male-to-male sexual contact, injection drug use, smoking, at-risk alcohol use, pre-ART viral load, year of ART initiation, ART regimen, CD4 count, clinical AIDS status, chronic hepatitis status, statin use, and study site. Solid line represents viral loads <20 copies/mL, dot-dashed line represents viral loads between 20 and 199 copies/mL, dashed line represents viral loads between 200 and 999 copies/mL, dotted line represents viral loads >999 copies/mL. (a) Crude; (b) Standardized.

The overall standardized risk of death without a cancer diagnosis, which was considered a competing risk in the analysis, was 12.2% (95% CI: 10.2%, 14.2%) (Table 4, Fig 2). The risk of death differed by viral load category, ranging from 10.7% among patients with viral loads under 20 copies/mL to 18.1% among patients with viral loads of at least 1,000 copies/mL six months after starting ART.

**Table 4:** Crude and standardized 10-year cumulative incidence, risk difference, and risk ratio estimates for death without a cancer diagnosis in 7,515 CNICS patients, averaged over 30 imputations.

	No. of events	No. of patients	Person- years	rude			Standardized ^a^		
				Risk, % (95% CI)	RD, % (95% CI)	RR (95% CI)	Risk, % (95% CI)	RD, % (95% CI)	RR (95% CI)
Total	560	7,515	40,110	12.7 (11.5, 13.9)			12.2 (10.2, 14.2)		
<20 cpm	196	4,281	22,392	8.99 (7.60, 10.4)	0	1	10.7 (8.18, 13.2)	0	1
20 to 199 cpm	112	1,694	9,625	11.5 (9.55, 13.5)	2.55 (0.34, 4.75)	1.28 (1.01, 1.55)	11.5 (9.11, 13.8)	0.74 (-1.68, 3.17)	1.07 (0.83, 1.30)
200 to 999 cpm	37	393	2,124	14.7 (9.55, 19.9)	5.73 (0.29, 11.2)	1.64 (0.99, 2.28)	15.0 (8.86, 21.1)	4.25 (-2.53, 11.0)	1.40 (0.73, 2.06)
>999 cpm	215	1,147	5,969	26.3 (22.9, 29.7)	17.3 (13.6, 21.0)	2.93 (2.31, 3.55)	18.1 (13.7, 22.5)	7.38 (2.40, 12.4)	1.69 (1.14, 2.24)

Abbreviations: CI, confidence interval; CNICS, Center for AIDS Research Network of Integrated Clinical Systems; cpm, copies per milliliter; RD, risk difference; RR, risk ratio.

^a^ Standardized estimates controlled for age, sex, race/ethnicity, male-to-male sexual contact, injection drug use, smoking, at-risk alcohol use, pre-ART viral load, year of ART initiation, ART regimen, CD4 count, clinical AIDS status, chronic hepatitis status, statin use, and study site.

## Discussion

Our objective was to evaluate 10-year cancer risk among HIV patients on antiretroviral therapy with low viral load under 1,000 copies/mL, while accounting for death from any cause as a competing event. Crude 10-year risk of first cancer diagnosis was highest for patients with viral loads between 200 and 999 copies/mL after six months of therapy, though this was largely due to imbalances in race, year of ART initiation, ART regimen, CD4 count, and study site; after controlling for these factors and other baseline confounders, there was no association between HIV RNA six months after ART initiation and first cancer risk.

Nearly 70% of all viral load observations used in the analysis fell below assay detection limits, which was expected given that the study population had been on ART for six months at baseline. Prior studies have typically replaced viral load observations below assay detection limits with a constant value, which can result in substantial bias, particularly when the proportion of censoring is high [26, 27]. In our previous study of total mortality in a similar sample of CNICS patients, using a simple substitution approach to account for left-censored viral loads resulted in violations of positivity, unstable weights, and upwardly biased hazard ratio estimates [19]. Here, we used nonparametric multiple imputation to account for left-censored viral loads. This approach allowed for the comparison of undetectable viral load observations collected over time using assays with different detection limits, without imposing assumptions about the underlying distribution of viral load, and likely resulted in less biased cumulative incidence estimates.

Studies that characterize cancer risk often measure incidence rates, typically expressed as the number of cancer events per 100,000 person-years, which assume that incident cancers occur at a constant rate over time. Here we estimated the probability of developing cancer over a specific 10-year period, which may provide a more intuitive measure of cumulative cancer risk. Additionally, a number of previous studies evaluating cancer trends among people with HIV have censored deaths in their analyses rather than using a competing risks approach. Treating deaths as censored observations (i.e., using the Kaplan-Meier survival function or standard Cox proportional hazards estimates) ignores the fact that HIV patients may die before being diagnosed with cancer, and will thereby overestimate cancer risk. While censoring competing events may not lead to significant bias when the risk of the competing event is rare, we observed nearly twice as many deaths as first cancer diagnoses (560 deaths vs. 290 cancer diagnoses) in our study sample of HIV patients on treatment. Moreover, censoring competing events may lead to additional bias when the risk of the competing event is differential by exposure, as was the case in this study. We expect that we arrived at less biased risk estimates by modeling the cumulative incidence function of cancer, while explicitly accounting for death from any cause without a cancer diagnosis as a competing risk.

We did not account for adherence, switching, or cessation of ART regimen in the analyses. For each treatment-naïve patient, we considered the first recorded date of concurrent prescription of three or more ART drugs as an indicator of starting a combination ART regimen, and ignored changes in treatment. We assumed that variables included in the analyses were measured without error, which is unlikely for self-reported behaviors such as tobacco, alcohol, and illicit drug use; however, we do not expect measurement error of confounders to be differential by exposure or outcome. Outcome misclassification was minimized in this study as cancer cases were confirmed through medical record review, and deaths verified using national and state death records.

Nearly a quarter of our study sample was lost to follow-up over the study period, and patients were followed for a median of five years. Given that the outcome of interest was 10-year cancer risk, it would be worthwhile to reassess our risk estimates after additional person-time has accumulated in the CNICS cohort. Due to the relatively small number of cancer cases observed in our study population, our estimates had limited precision and we were unable to make inferences about the potential relationship between incomplete viral suppression and specific cancer types. Therefore, pooling data from other clinical cohorts to verify and expand upon our results is warranted. Nevertheless, we expect that the results of this study are generalizable to HIV patients receiving care and treatment at academic medical centers in the US, and we observed clinically meaningful trends that highlight potential avenues for increased and/or early cancer screening and prevention among people living and aging with HIV. In this study, non-Hodgkin lymphoma, Kaposi sarcoma, lung cancer, Hodgkin lymphoma, prostate cancer, anal cancer, breast cancer, and liver cancer were the most commonly observed cancer types, consistent with prior studies of cancer in HIV patients after the introduction of ART [1, 3, 28]. This has implications for targeted cancer screening for HIV patients, including those on successful ART, as well as preventive interventions such as smoking cessation programs and human papillomavirus and hepatitis B vaccination. We also observed a higher proportion of Hodgkin lymphoma cases among patients with lower viral loads, possibly related to immune reconstitution [29].

We observed a 10-year standardized first cancer risk of 6.9% in our sample of HIV patients after starting therapy, which provides support for existing evidence that cancer poses a significant threat to HIV patients after ART initiation, even among patients who have rapidly achieved viral suppression. Exposure status was based on a single HIV RNA measurement collected approximately six months after ART initiation, as we considered viral suppression at this time point a relevant marker of early treatment success. However, a single detectable viral load measurement could represent either a transient increase in viral load or sustained low-level viremia. While we did not observe an association between early virologic response and subsequent cancer risk in this study, using longitudinal viral load measurements to assess exposure is warranted to better understand the dynamic nature of HIV RNA suppression. Continued research on the role of low-level circulating HIV RNA on cancer development is of particular importance, as occurrences of incomplete viral suppression will be observed more frequently in clinical practice with more people with HIV gaining access to effective ART in the current era of universal treatment.

## Supporting information

S1 Fig Distribution of viral loads up to 200 copies/mL for 7,515 CNICS patients, six months after ART initiation. Dotted line indicates 20 copies/mL. (a) After nonparametric multiple imputation of left-censored viral load observations, averaged over 30 imputations of original dataset; (b) After substitution of left-censored viral load observations with half of detection limit of viral load assay.
(TIF)
