# Supplementary figures and images for: Cancer risk in HIV patients with incomplete viral suppression after initiation of antiretroviral therapy
Source: PLoS One. 2018 Jun 5;13(6):e0197665. doi: 10.1371/journal.pone.0197665 (PMC5988275; doi:10.1371/journal.pone.0197665)

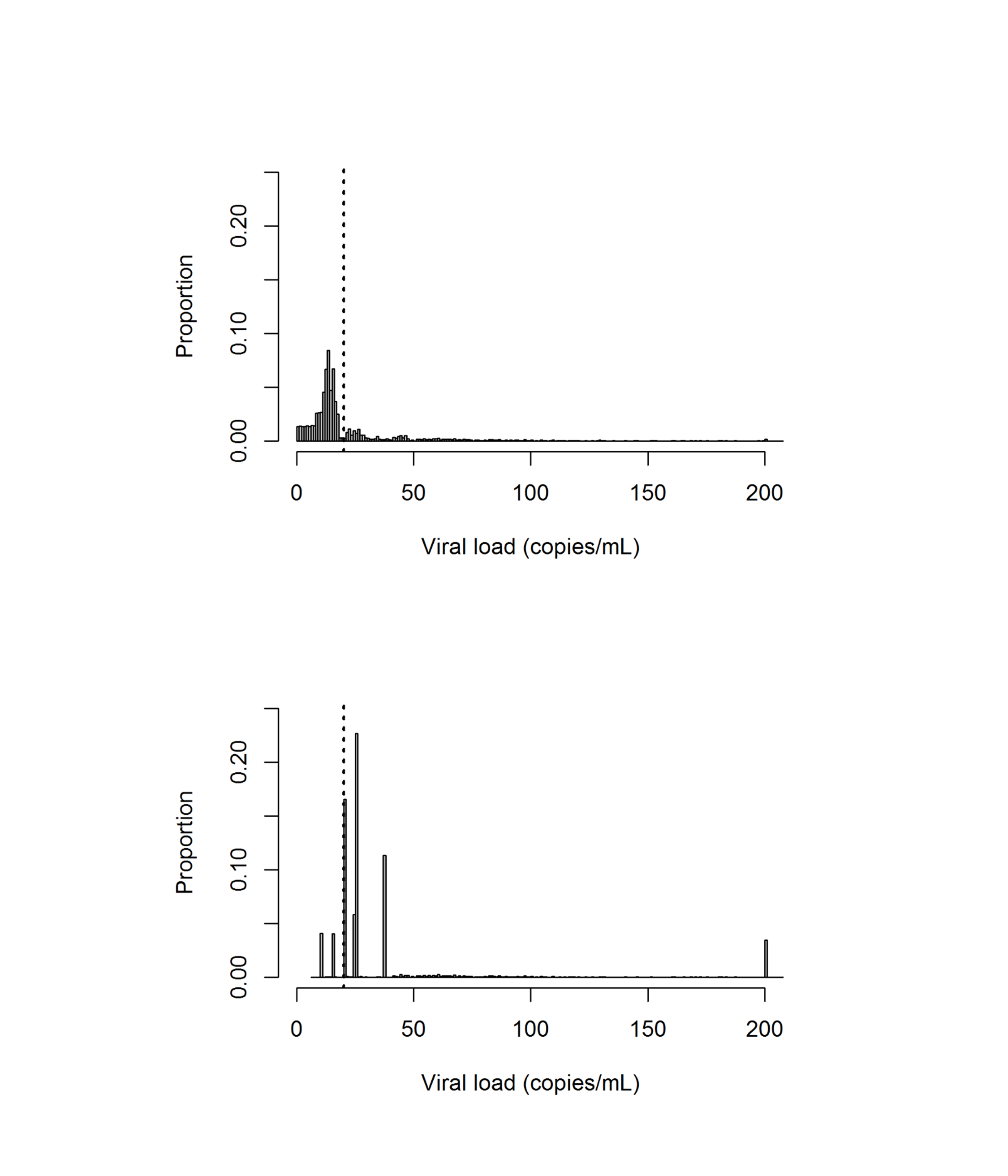

Supplement: S1 Fig — Dotted line indicates 20 copies/mL. (a) After nonparametric multiple imputation of left-censored viral load observations, averaged over 30 imputations of original dataset; (b) After substitution of left-censored viral load observations with half of detection limit of viral load assay. (TIF) [file pone.0197665.s001.tif]
